# Persistence and size of seasonal populations on a consumer–resource relationship depends on the allocation strategy toward life-history functions

**DOI:** 10.1038/s41598-020-77326-1

**Published:** 2020-12-08

**Authors:** Rodrigo Gutiérrez, Fernando Córdova-Lepe, Felipe N. Moreno-Gómez, Nelson A. Velásquez

**Affiliations:** 1 Doctorado en Modelamiento Matemático Aplicado, Facultad de Ciencias Básicas, Universidad Católica del Maule, Talca, Chile; 2 Laboratorio de Comunicación Animal, Departamento de Biología y Química, Facultad de Ciencias Básicas, Universidad Católica del Maule, Talca, Chile; 3 Facultad de Ciencias Básicas, Departamento de Matemática, Física y Estadística, Universidad Católica del Maule, Talca, Chile; 4 Laboratorio de Bioacústica y Ecología del Comportamiento Animal, Departamento de Biología y Química, Facultad de Ciencias Básicas, Universidad Católica del Maule, Talca, Chile

**Keywords:** Ecological modelling, Population dynamics

## Abstract

The long-term ecological dynamics of a population inhabiting a seasonal environment is analyzed using a semi-discrete or impulsive system to represent the consumer–resource interaction. The resource corresponds to an incoming energy flow for consumers that is allocated to reproduction as well as to maintenance in each non-reproductive season. The energy invested in these life-history functions is used in reproductive events, determining the size of the offspring in each reproductive season. Two long-term dynamic patterns are found, resulting in either the persistence or the extinction of the population of consumers. In addition, our model indicates that only one energy allocation strategy provides an optimal combination between individual consumption and long-term population size. The current study contributes to the understanding of how the individual-level and the population-level are interrelated, exhibiting the importance of incorporating phenotypic traits in population dynamics.

## Introduction

The quality and quantity of resources available for consumption are key factors influencing the abundance dynamics of populations: they affect the extent of intraspecific competition and impose upper limits on the population abundance^1,2^. In addition, the availability of resources is likely to affect life history traits, such as growth, reproduction, and survival, which will ultimately also affect population growth^3^. Understanding how organisms allocate their limited resources toward vital functions necessitates the determination of the different traits that define their life-history strategies, and which are expected to evolve in such a way as to maximize fitness^4^. The allocation strategies that have been studied to determine the optimal life-history traits^5–7^ that allow maximizing the population growth rate are considered in the Lotka–Euler equation or through its approximation, the net reproductive rate ${\mathscr {R}}_0$ (i.e., the total number of offspring that an individual produces during its lifetime^3,5,7,8^). However, few studies have considered the interaction between consumers and resources within a broad ecological approximation, incorporating phenotypic traits such as the energy allocation strategy to understand its effects on population dynamics^9,10^.

Consumer–resource interactions are one of the most important intraspecific relationships in population ecology, with an extensive research agenda encompassing the prey-predator, plant-herbivore, and host-parasite systems^11,12^. Furthermore, these interactions are an underlying component of any food web^13^. For instance, DeSiervo et al. (2020) using this approach and an experimental model, investigate whether the population dynamics of the Arctic mosquito species, *Aedes nigripes*, is controlled by its food (aquatic biofilms), or its predators (diving beetles). Using the traditional differential equation approach for modeling the population dynamics, Bideault et al. (2019) studied the effects of temperature on the consumer resource interaction strength^14^, whose results are highly relevant under the current global warming scenario.

One of the main objectives of population ecology is to describe and determine the changes in the number of individuals that exploit a particular resource over time is, which is frequently done through a theoretical approach using consumer–resource models^15^. In recent years, new mathematical modeling approaches have been formulated and used to address new ways of representing the consumer–resource relationship. For instance, the Integral Projection Models (IPM) are adjustment models that estimate the survival, growth, and reproduction rate, allowing the description of how a population structured by variable and continuous states at the individual level (e.g., body size) changes in discrete time^16–18^. Similarly, Individual-Based Models (IBM), which were developed through computer programming, pursue the determination of the impact of a phenotype on the population dynamics and the strategies that emerge as a product of abiotic factors^19–21^. These modeling approaches have allowed a deeper understanding of the interaction between individual traits and population dynamics^22,23^.

The incorporation of physiological and energetic principles at the individual level enables the emergence of dynamic patterns at the population level. Thus, the theory of Dynamic Energy Storage (DEB)^24^ and the Metabolic Theory of Ecology (MTE)^25,26^ provide a framework focused at the individual level. For their formulation, both these theories consider environmental variables and physiological principles related to the optimal acquisition, ingestion, and resource allocation towards the functions of reproduction and somatic growth, which are essential components for the implementation of both IPM and IBM. 

Among the mathematical models representing the consumer–resource relationship based on ordinary differential equations^27–29^ there stand out both biomass conversion (BC) and individual survival (IS) models. These models differ in the representation of the per capita population growth rate of the consumers^30^.The first assumes a rate dependent on the consumption of resources, therefore relating the decrease in the density of resources with the increase in consumer biomass. The second considers a rate dependent on the density of consumers restricted by intraspecific interactions (e.g., in the logistic model, the per capita population growth rate is inversely proportional to the intraspecific competition factor). It is important to note that for both models, the life-history traits of the organisms are a common underlying component in the formulation of the per capita population growth rate (e.g., predation efficiency, prey search and management time, the average number of progeny, probability of survival until some specific age).

The classical approach to the mathematical modeling of biological systems provided by differential (or difference) equations has considered the resource allocation to different functions of life history (e.g., growth and reproduction) to predict optimal life-history traits (e.g., age and body size of maturity)^5,6,31^, and also to determine the optimal strategies that maximize the lifetime reproductive success of individuals. However, these results, focused on the individual level, have not been scaled at a population level: in particular, they ignore the influence of energy resource allocation on population abundance. Thus, Akhmetzhanov et al. (2011)^10^ analyzed the allocation energy strategies for reproduction and resource foraging, using a mathematical approach with a semi-discrete model^32^. The long-term dynamics that emerge from the proposed model include the extinction of the population due to an over-exploitation of resources, coexistence in a season-to-season equilibrium, and oscillations in population size and resources. Furthermore, they concluded that the optimal allocation strategies have a stabilizing role in the consumer–resource interaction. However, no link has been established between allocation strategies towards different life-history functions and long-term population abundance.

Considering that the emergence of dynamic patterns are linked to how individuals allocate their resources to different life-history functions, our objective is to analyze the consumer–resource relationship and its effects on the persistence of populations living in seasonal environments, incorporating a constant strategy of energy allocation towards both reproduction and maintenance. To address our objective, we use semi-discrete models, due to the division of the annual cycle into two seasons (namely, reproductive and non-reproductive) acting on two different time scales (namely, continuous and discrete). These models have a common mathematical formalism in terms of impulsive differential equations^33,34^, which are widely used to address topics of interest in epidemiology and population ecology^35–39^. Our consumer–resource model has a “bottom-up” mechanistic formulation^40–42^ to obtain a better understanding of the dynamic behaviors of the populations by incorporating the individual allocation of energetic resources towards reproduction.

## Consumer–resource and energetic model

Our consumer–resource and energetic model considers a population of individuals with generational overlap, in which consumers feed exclusively on a resource that is completely restored every cycle. In addition, individuals experience intraspecific competition for resources. The annual cycle is divided into two seasons: reproductive and non-reproductive. Throughout each non-reproductive season, individuals consume the resource at a rate dependent on the equal division of the resource (ratio-dependent), which is allocated between reproduction and maintenance.

In each temporal cycle of length $\tau > 0$, the reproductive season represents a relatively small period^43^, which is symbolized by instants $t_ {n} = n\tau$, $n \ge 0$. Thus, the development of non-reproductive seasons are taken to be in the intervals $(n\tau , (n + 1)\tau ]$.

### Dynamics in the non-reproductive season

Let *P*(*t*) be a measure of the population abundance at time $t \ge 0$, which decreases by natural deaths in each non-reproductive season, according to the Malthus law with rate $\lambda >0$. Individuals are exclusive consumers of a resource whose density is denoted by *R*(*t*). The individual consumption (per unit time) ${\mathscr {C}}$ increases with respect to the resource per capita rate *R*(*t*) /*P*(*t*)^44,45^ where $R_{max}>0$ is the maximum rate of consumption in the presence of a high resource density per individual. Then,

1 $$\begin{aligned} {\mathscr {C}}(t)=R_{max}\dfrac{R(t)/P(t)}{r_0 + R(t)/P(t)}=\dfrac{R_{max}R(t)}{r_0 P(t)+R(t)}, \end{aligned}$$

where $$ r_0 $$ is the half-saturation constant, namely, the per capita resource density for which an individual consumption rate, equal to $$R_ {max}/2 $$, is obtained. Therefore, the resource consumed by the population changes at a rate that is described by

$$\begin{aligned} R'(t)=-{\mathscr {C}}(t)P(t),\quad t\in (n\tau ,(n+1)\tau ]. \end{aligned}$$

The rate (1) is the ratio-dependent functional response. This rate is a Holling type II functional response with the resource per capita rate *R*(*t*) /*P*(*t*) as variable^44^. The use of the ratio-dependent functional response in predator–prey models provides the simulation of more realistic scenarios with a greater dynamic richness. In addition, it corrects the assumption that predation rates are unsaturated and invalidates the paradoxes of biological control and enrichment^29,46,47^.

The resources consumed by each individual are an energy source that is assigned to reproduction and maintenance in the fraction $\alpha$ ($0<\alpha <1$) and $1-\alpha$, respectively. The energy invested in reproduction and maintenance are quantified by $E_r (t)$ and $E_ {m} (t)$ at time $t\ge 0$, respectively.

During the non-reproductive season, the individuals forage, obtaining the energy necessary for their maintenance. In foraging, the associated costs can be measured in terms of time and energy spent, without forgetting that vital processes have intrinsic costs^4^. In our mathematical approach, these costs (per unit time) are described by the fraction of the resource (such as energy) which is invested in maintenance:

2 $$\begin{aligned} C_{m}(t)=(1-\alpha )\xi {\mathscr {C}}(t),\quad 0<\xi <1. \end{aligned}$$

Thus, the energy reproduction and maintenance rates are determined by

3 $$\begin{aligned} E_{r}'(t)=\alpha {\mathscr {C}}(t)\quad \text {and}\quad E_{m}'(t)=(1-\alpha )\xi _{c}{\mathscr {C}}(t),\quad t\in (n\tau ,(n+1)\tau ], \end{aligned}$$

where $\xi _{c}=1-\xi$. Importantly, $E_m$ corresponds to the energy that will be principally used by the organism to maintain its offspring in each reproductive event, not to maintain itself. Indeed, for the sake of simplicity, the mortality rate $\lambda$ is taken to be independent of $E_m$.

**Figure 1:**
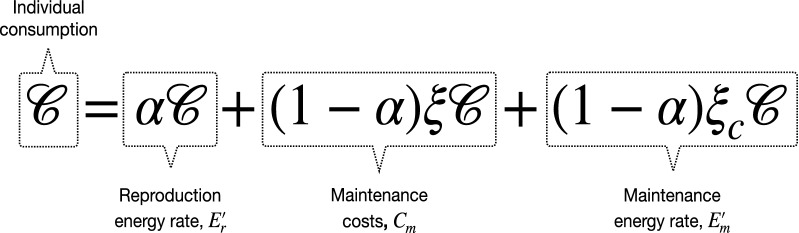
Relationships among individual consumption (per unit time), maintenance costs (per unit time), reproduction, and maintenance energy rates.

Figure 1, illustrates the relationships among the rates (1), (2) and (3).

### Dynamics in the reproductive season

Reproduction is an essential process in the life of organisms. This is evidenced by the evolution of the different life-histories leading to the maximization of reproductive success. However, since each organism has a finite number of resources, when more energy is invested in reproduction, then less energy is invested in other functions, implying a compromise between the future reproduction and survival of organisms^48^. According to Stearns (1989), the most important trade-off in the life-history of organisms usually involves reproduction costs, composed of both fecundity and survival costs.

The fecundity, defined as the potential maximum of the physiological reproductive performance of an individual throughout its useful life, is a concept widely studied in population ecology^49^. The current reproductive performance of organisms is described by fertility, which can vary spatially and temporally among individuals due to variation of both environmental conditions and demographics^49^. Thus, considering that the consumed resource is constituted of energy for individuals, we assume a fertility ${\mathscr {B}}$ proportional to the reproductive energy, namely ${\mathscr {B}}(E_{r} (n \tau )) = \gamma E_{r} (n \tau )$, where $\gamma$ is the number of offspring per unit of reproductive energy. According to Reznick (1992), the cost of reproduction is represented by an inverse relation between high fertility and both survival or future reproduction of organisms, which in energetic terms, could imply a reduction in the energy available for maintenance and reproduction due to payment for the costs of survival and fecundity. To represent the fecundity and survival costs, we will consider both increasing and saturated expressions with respect to the increasing fertility, described respectively by $C_{f} = \kappa {\mathscr {B}}$ with $\kappa =\kappa (\gamma )>0$ such that $0<\kappa \gamma \le 1$ and $C_ {s} = \{{\mathscr {B}} / ({\mathscr {B}}_0 + {\mathscr {B}}) \} E_{m} (n \tau )$, where ${\mathscr {B}}_0$ is the number of offspring at which survival costs reaches one-half of $E_{m} (n \tau )$. Therefore, $E_{r} (n \tau ^+) = E_{r} (n \tau ) -C_{f}$ and $E_{m} (n \tau ^+) = E_{m} (n \tau ) -C_{s}$, or equivalently

$$\begin{aligned} E_{r}(n\tau ^+)=E_{r}(n\tau )-\kappa \gamma E_{r}(n\tau ) = (1-\kappa \gamma )E_{r}(n\tau ),\quad \text {and}\quad E_{m}(n\tau ^+)=E_{m}(n\tau )-\dfrac{{\mathscr {B}}}{{\mathscr {B}}_0 + {\mathscr {B}}}E_{m}(n\tau ) = \dfrac{{\mathscr {B}}_{0}E_{m}(n\tau )}{{\mathscr {B}}_0+\gamma E_{r}(n\tau )}. \end{aligned}$$

Note that $\kappa \gamma$ is a non-dimensional constant, which corresponds to the fraction of reproductive energy used in the reproductive events, denominated reproductive effort. Consequently, the fertility costs $C_f$ are a fraction of the reproduction energy $E_{r}(n\tau )$.

The per capita growth rate *r* is defined to be the product of the fertility ${\mathscr {B}}$ and the probability of surviving the reproductive season ${\mathscr {S}}$^15^, which is dependent on the energy allocated to reproduction and maintenance (after the application of costs), respectively. Then,

$$\begin{aligned} r={\mathscr {B}}(E_{r}(n\tau ))\cdot {\mathscr {S}}(E_{m}(n\tau ^+)), \end{aligned}$$

with ${\mathscr {S}}(x) = x / (e_ {1/2} + x)$ where $e_ {1/2}$ is the energy at which the survival probability reaches one-half^50,51^. In addition, we assume that a fraction $\mu$ ($0<\mu <1$) of the individuals that reproduce die during the reproductive season. Then, the population abundance post-reproductive season is

$$\begin{aligned} P(n\tau ^+)=(1-\mu )P(n\tau )+\gamma E_{r}(n\tau )\dfrac{E_{m}(n\tau ^+)}{e_{1/2}+E_{m}(n\tau ^+)}P(n\tau ). \end{aligned}$$

Finally, at the end of each reproductive season, the resource density is restored to the value $R(n\tau ^+) = K$.

### The model

The dynamics are modeled by a consumer–resource model which divides the annual cycle into two seasons and posits a constant allocation of the resource, as an energy source, towards life-history functions. The relations between energy states, resource density, and population abundance allow defining the following impulse differential system:

4 $$\begin{aligned} X_{\eta }:\left\{ \begin{array}{lll} \left. \begin{array}{lcl} R'(t){}={}-{\mathscr {C}}(t)P(t)\\ E_{r}'(t){}={}\alpha {\mathscr {C}}(t)\\ E_{m}'(t){}={}(1-\alpha )\xi _{c}{\mathscr {C}}(t)\\ P'(t){}={}-\lambda P(t) \end{array} \right\} \quad \text {if} \quad t\in (n\tau ,\, (n+1)\tau ]\,,\\ \left. \begin{array}{lcl} R(t^{+}){}={}K\\ E_{r}(t^{+}) {} = {}(1-\kappa \gamma )E_{r}(t)\\ E_{m}(t^{+}) {} = {}\dfrac{{\mathscr {B}}_{0}E_{m}(t)}{{\mathscr {B}}_0+\gamma E_{r}(t)}\\ P(t^{+}){}={}\left[ 1-\mu +\gamma E_{r}(t)\dfrac{E_{m}(t^+)}{e_{1/2}+E_{m}(t^+)}\right] P(t) \end{array} \right\} \quad \text {if} \quad t=n\tau , \end{array} \right. \end{aligned}$$

with $\eta =(R_{max},r_0,\tau ,\lambda ,K,{\mathscr {B}}_0,\gamma ,\kappa ,e_{1/2},\xi ,\alpha ,\mu )\in {\mathbb {R}}_{+}^{9}\times (0,1)^3$ such that $0<\kappa \gamma \le 1$, a set of parameters with different ecological meanings. The variation of the individual’s internal energy $E(t)=E_{r}(t)+E_{m}(t)$ is described by

$$\begin{aligned} E'(t) = \{\alpha +(1-\alpha )\xi _{c}\}\left\{ \lambda \dfrac{R(t)}{P(t)}-\left( \dfrac{R( t)}{P (t)}\right) '\right\} , \quad t \in (n \tau , (n + 1) \tau ], \end{aligned}$$

where the influence of the per capita rate of resource on the individual’s energy and the link between individual and population level can be observed. Integrating the previous equation we have the individual’s internal energy in each non-reproductive season is

$$\begin{aligned} E(t) =E(n\tau ^+) + \{\alpha +(1-\alpha )\xi _{c}\}\Phi (t,R(t),P(n\tau ^+)), \end{aligned}$$

where

5 $$\begin{aligned} \Phi (t,R(t),P(n\tau ^+))=\frac{K-R(t)e^{\lambda (t-n\tau )}}{P(n\tau ^+)}+\dfrac{\lambda }{P(n\tau ^+)}\int _{n\tau }^{t}{R(s)e^{\lambda (s-n\tau )}ds}. \end{aligned}$$

## Results

The long-term population dynamics (i.e., extinction or persistence in an equilibrium value) of the system (2) is mainly dependent on the constant allocation of energy between life-history functions. In the case of population persistence, there is a unique long-term allocation strategy towards reproductive functions that maximizes the population abundance and minimizes individual consumption. In addition, this strategy is dependent on the parameters associated with both fertility ($\kappa$ and ${\mathscr {B}}_0)$ and survival $(\xi _{c}$ and $e_{1/2}$) costs.

### Preliminary results

To investigate long-term dynamic patterns, we will relate the energy states of reproduction and maintenance, resource density and population abundance at the end of reproductive seasons, namely in the time sequence $\{n\tau ^+\}_{n\ge 0}$. In the system (4) the relationships between the state variables are the same for each $\tau$ unit of time. Then, there is a transformation that relates to the vector $(R,E_ {r}, E_ {m},P) ((n + 1)\tau ^+)$ with $(R,E_ {r}, E_ {m}, P) (n \tau ^+)$ such that $(n + 1) \tau ^+ - n\tau ^+ = \tau$. This relationship is determined by the following discretization (or stroboscopic map) of the impulsive differential system (4):

6 $$\begin{aligned} \left\{ \begin{array}{lll} E_{r}((n+1)\tau ^+)&=(1-\kappa \gamma )[E_{r}(n\tau ^+)+\alpha \Phi (n,P(n\tau ^+))],\\ &{}&{}\\ E_{m}((n+1)\tau ^+)&=\dfrac{{\mathscr {B}}_0[E_{m}(n\tau ^+)+(1-\alpha )\xi _{c}\Phi (n,P(n\tau ^+))]}{{\mathscr {B}}_0+\gamma [E_{r}(n\tau ^+)+\alpha \Phi (n,P(n\tau ^+))]},\\  P((n+1)\tau ^+)&=\left\{ 1-\mu +\dfrac{\gamma [E_{r}(n\tau ^+)+\alpha \Phi (n,P(n\tau ^+))]E_{m}((n+1)\tau ^+)}{e_{1/2}+E_{m}((n+1)\tau ^+)}\right\} P(n\tau ^+)e^{-\lambda \tau } \end{array} \right. \end{aligned}$$

and $R(n\tau ^+)=K$, where the function $\Phi$ defined by (5) is evaluated at $t=(n+1)\tau$ and extended to $p=0$:

7 $$\begin{aligned} \Phi (n,p)=\left\{ \begin{array}{ll} \dfrac{R_{max}(e^{\lambda \tau }-1)}{\lambda }, &\quad \text {if}\, p=0, \\ &{}\\ \dfrac{K-R((n+1)\tau )e^{\lambda \tau }}{p}+\dfrac{\lambda }{p}\displaystyle \int _{n\tau }^{(n+1)\tau }{R(s)e^{\lambda (s-n\tau )}ds}, & \quad \text {if}\, p\ne 0, \end{array} \right. \end{aligned}$$

for any $$n\ge 0$$. Indeed, in non-reproductive seasons the consumer–resource dynamics are described by the continuous component of the system (4), which can be solved. Directly we have $$P(t)=P(n\tau ^+)e^{-\lambda (t-n\tau )}$$ for any $$t\in (n\tau ,(n+1)\tau ]$$. In addition, the per capita consumption rate (1) can be expressed in terms of $$ R'(t)$$. Therefore, reproductive and maintenance energy rates are determined by

$$\begin{aligned} E_r'(t)=-\alpha \frac{R'(t)e^{\lambda (t-n\tau )}}{P(n\tau ^+)}\quad \text {and}\quad E_m'(t)=-(1-\alpha )\xi _{c}\frac{R'(t)e^{\lambda (t-n\tau )}}{P(n\tau ^+)},\quad t\in (n\tau ,(n+1)\tau ]. \end{aligned}$$

Integrating these functions in the interval $(n \tau , t]$, we obtain

8 $$\begin{aligned} E_r(t)&=  {} E_{r}(n\tau ^+)+\alpha \Phi (t,R(t),P(n\tau ^+)) \end{aligned}$$

9 $$\begin{aligned} E_m(t)&=  {} E_m(n\tau ^+)+(1-\alpha )\xi _{c}\Phi (t,R(t),P(n\tau ^+)). \end{aligned}$$

Evaluating Eqs. (8)–(9) at $t = (n + 1)\tau$ (end of the non-reproductive season), we have

10 $$\begin{aligned} E_r((n+1)\tau )&=  {} E_{r}(n\tau ^+)+\alpha \Phi (n,P(n\tau ^+)), \end{aligned}$$

11 $$\begin{aligned} E_m(n+1)\tau )&=  {} E_m(n\tau ^+)+(1-\alpha )\xi _{c}\Phi (n,P(n\tau ^+)), \end{aligned}$$

with $\Phi (n,P(n\tau ^+))= \Phi ((n+1)\tau ,R((n+1)\tau ),P(n\tau ^+))$ and $P((n+1)\tau )=P(n\tau ^+)e^{-\lambda \tau }$ for any $n\ge 0$. In addition, evaluating the discrete component of system (4) at $t=(n+1)\tau$, we obtain

12 $$\begin{aligned} E_{r}((n+1)\tau ^+)&=  {} (1-\kappa \gamma )E_{r}((n+1)\tau ), \end{aligned}$$

13 $$\begin{aligned} E_m((n+1)\tau ^+)&=  {} \frac{{\mathscr {B}}_0E_m((n+1)\tau )}{{\mathscr {B}}_0+\gamma E_r((n+1)\tau )}, \end{aligned}$$

14 $$\begin{aligned} P((n+1)\tau ^+)= & {} \left[ 1-\mu +\gamma E_{r}((n+1)\tau )\frac{E_{m}((n+1)\tau ^+)}{e_{1/2}+E_{m}((n+1)\tau ^+)}\right] P((n+1)\tau ). \end{aligned}$$

Finally, substituting the Eqs. (10)–(11) into the Esq. (12)–(14) we obtain the discretization given by Eq. (6).

In order to obtain the equilibrium points of system (6), we can solve the following equations:

$$\begin{aligned} e_{r}&=  {} (1-\kappa \gamma )[e_{r}+\alpha \Phi (\rho )],\\ e_{m}&=  {} \dfrac{{\mathscr {B}}_{0}[e_{m}+(1-\alpha )\xi _{c}\Phi (\rho )]}{{\mathscr {B}}_0+\gamma [e_{r}+\alpha \Phi (\rho )]},\\ \rho&=  {} \left\{ 1-\mu +\gamma [e_{r}+\alpha \Phi (\rho )]\dfrac{e_{m}}{e_{1/2}+e_{m}}\right\} \rho e^{-\lambda \tau }, \end{aligned}$$

where $e_{r}:=\lim _{n\rightarrow +\infty }{E_{r}(n\tau ^+)}$, $e_{m}:=\lim _{n\rightarrow +\infty }{E_{m}(n\tau ^+)}$, $\rho :=\lim _{n\rightarrow +\infty }{P(n\tau ^+)}$ and then, $\Phi (\rho ):=\lim _{n\rightarrow +\infty }{\Phi (n,P(n\tau ^+))}$. From the first equation, we have $e_{r}=(1-\kappa \gamma )\alpha \Phi (P)/\kappa \gamma$ and then $e_{m}=\kappa {\mathscr {B}}_0 (1-\alpha )\xi _{c}/\alpha$ for $P\in \{0,\rho \}$ such that $\Phi (0)=R_{max}(e^{\lambda \tau }-1)/\lambda$ and

15 $$\begin{aligned} \Phi (\rho )={\mathscr {A}},\quad \text {where}\quad {\mathscr {A}}=\dfrac{e^{\lambda \tau }-1+\mu }{{\mathscr {B}}_0}\cdot \left\{ \dfrac{e_{1/2}\xi _{c}^{-1}}{1-\alpha } +\dfrac{\kappa {\mathscr {B}}_0}{\alpha }\right\} . \end{aligned}$$

Therefore, assuming $\lambda =0$, we have $\Gamma (\rho )=K-{\mathscr {A}}\rho$ (from equations (7) and (15)) where $\Gamma :=\lim _{n\rightarrow +\infty }{R((n+1)\tau )}$, $\rho$ is the solution of

$$\begin{aligned} r_0\ln \left( \dfrac{K-{\mathscr {A}}\rho }{K}\right) -{\mathscr {A}}=-R_{max}\tau , \end{aligned}$$

if, and only if,

16 $$\begin{aligned} \rho =\dfrac{K}{{\mathscr {A}}}\left[ 1-\exp \left( \dfrac{{\mathscr {A}}-R_{max}\tau }{r_0}\right) \right] . \end{aligned}$$

### Long-term population dynamics

**Figure 2:**
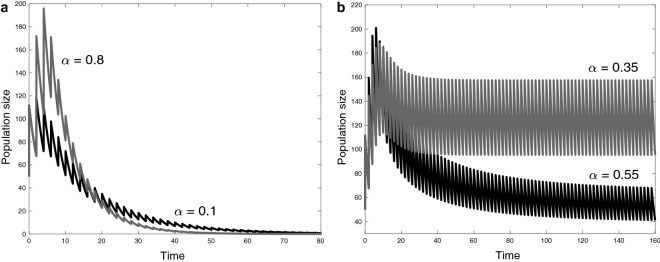
Long-term behavior of solutions of the model (4). Peak values correspond to the solution of the discrete model (6) in its population component. (**a**) Extinction behavior, considering $\alpha \in \{0.1,\,0.8\}$ as energy allocation strategy toward reproductive and (**b**) persistence behavior considering $\alpha \in \{0.35,\, 0.55\}$. We consider the following parameter set $\eta =(2,0.5,2,0.25,500,2,1,0.5,0.5,0.86,\alpha ,0.1)$ where the constant of fertility costs is described by $\kappa =(1+\gamma )^{-1}$.

From the discretization (6), there are two dynamic behaviors for the long-term population abundance: extinction (see Fig. 2a) and persistence (see Fig. 2b).

The differentiation of these behaviors strongly depends on the individual consumption of resources defined throughout each non-reproductive season by

$$\begin{aligned} C_{I}(t) = \frac{[K-R(t)]e^{\lambda (t-n\tau )}}{P(n\tau ^+)},\, t\in (n\tau ,(n+1)\tau ]. \end{aligned}$$

At the end of each non-reproductive season, namely at the time $t = (n + 1) \tau$, the individual consumption is given by

17 $$\begin{aligned} C_I((n+1)\tau ) = \frac{[K-R((n+1)\tau )]e^{\lambda \tau }}{P(n\tau ^+)}, \end{aligned}$$

where $R((n+1)\tau )$ is the non-consumed resource density by the population, the amount that is obtained from the implicit solution of the resource density equation in the continuous component of the system (4). Thus, projecting the individual consumption of the resource into the long term, and taking $\Gamma (\rho )=(K-{\mathscr {A}}\rho +\lambda {\mathscr {I}})e^{-\lambda \tau }$, the expression (17) assumes the form

18 $$\begin{aligned} C_{I}^{\infty }(\rho )=\left\{ \begin{array}{ll} {\mathscr {A}}+\dfrac{K(e^{\lambda \tau }-1)-\lambda {\mathscr {I}}}{\rho },&{}\quad \text {if}\, \rho \ne 0,\\ &{}\\ \dfrac{R_{max}(e^{\lambda \tau }-1)}{\lambda },&{}\quad \text {if}\, \rho =0, \end{array}\right. \end{aligned}$$

where ${\mathscr {I}}:=\lim _{n\rightarrow +\infty }{\int _{n\tau }^{(n+1)\tau }{R(s)e^{\lambda (s-n\tau )}ds}}$. On the one hand, the individual consumption $C_{I}^{\infty }$ is composed of a basis amount corresponding to the term ${\mathscr {A}}$ and an amount resulting from the equal division of a resource not consumed by individuals dying during the non-reproductive season, $K(e^{\lambda \tau } -1) - \lambda {\mathscr {I}}$. Furthermore, when the population experiences a reduced mortality during the non-reproductive season (i.e., $\lambda \approx 0)$, the individual consumption is $C_{I}^{\infty } (\rho ) \approx {\mathscr {A}}$. On the other hand, whether the population abundance is low, the per capita resource is high, implying an individual consumption close to $R_ {max} (e^{\lambda \tau } -1) / \lambda$ (equivalent to taking the limit of $C_{I}((n+1)\tau )$ as $P(n\tau ^+)\rightarrow 0$). Certainly, this quantity does not represent the effective individual consumption, but rather establishes an upper limit for this and therefore represents a value of non-persistence. Thus, behaviors related to the equilibrium solutions of the discrete system (6) can be differentiated by the threshold value

$$\begin{aligned} {\mathscr {U}}=\dfrac{C_{I}^{\infty }(0)}{C_{I}^{\infty }(\rho )}. \end{aligned}$$

In particular, when the mortality of the population is low, the long-term abundance is described by Eq. (16) and the threshold value assumes the following form ${\mathscr {U}}=R_{max}\tau /{\mathscr {A}}$. Thus, we conclude that the persistence of the population is established when ${\mathscr {U}}>1$ and extinction when ${\mathscr {U}}\le 1$.

Finally, we can see that the stabilization of population size in the long term is in response to a dense-dependent behaviour where the per capita growth rate in the long term is $r_{\infty }:=\{\alpha {\mathscr {A}}/\kappa \}{\mathscr {S}}(e_m)$ equivalent to mortality fraction $\mu$, where $e_{m}=\kappa {\mathscr {B}}_{0}(1-\alpha )\xi _{c}/\alpha$ is the equilibrium value of energy maintenance after the reproductive season. In addition, the derivative of *r* with respect to *P* is

$$\begin{aligned} \dfrac{dr}{dP} = \gamma \dfrac{d\Phi }{d P}\left\{ \alpha {\mathscr {S}}(E_{m})+(E_{r}+\alpha \Phi )\dfrac{d{\mathscr {S}}}{dE_{m}}\cdot \dfrac{dE_{m}}{d\Phi }\right\} , \end{aligned}$$

where $d\Phi /d P<0$, $dr/d P<0$ and $dr/d\Phi >0$ are obtained, which explains the expected dense-dependency.

Then, we can derive the following conclusion.

#### **Theorem 1**

*We consider the threshold value* ${\mathscr {U}}=R_{max}\tau /{\mathscr {A}}$.

1. *If* ${\mathscr {U}}\le 1$ *then the long-term population behavior is extinction.*
2. *If* ${\mathscr {U}}>1$ *then the long-term population behavior is persistence.*

#### *Proof*

We divide the proof into two cases: $\kappa \gamma =1$ and $0<\kappa \gamma <1$. In the first case we define the functions

$$\begin{aligned} G(e_{m},p)&= \frac{{\mathscr {B}}_0[e_{m}+(1-\alpha )\xi _{c}\Phi (p)]}{{\mathscr {B}}_{0}+\gamma \alpha \Phi (p)},\\ H(e_{m},p) &=  \left\{ 1-\mu +\gamma \alpha \Phi (p)\frac{G(e_{r},e_{m},p)}{e_{1/2}+G(e_{r},e_{m},p)}\right\} p. \end{aligned}$$

Therefore, the discrete system defined by equations $e_{m}^{k+1}=G(e_{m}^{k},p^{k})$ and $p^{k+1}=H(e_{m}^{k},p^{k})$ with $k\ge 0$ has two equilibrium solutions: ${\mathscr {E}}(P)=(E_{m},P)$ where $E_{m}={\mathscr {B}}_0 (1-\alpha )\xi _{c}/\alpha \gamma$ and $P\in \{0,\rho \}$. Thus,

$$\begin{aligned} \dfrac{\partial G}{\partial e_m}&=  {} \dfrac{{\mathscr {B}}_0}{{\mathscr {B}}_0+\gamma \alpha \Phi (p)}, \quad \dfrac{\partial G}{\partial p}=\dfrac{{\mathscr {B}}_0[{\mathscr {B}}_0(1-\alpha )\xi _{c}-\alpha \gamma e_{m}]\Phi '(p)}{\{{\mathscr {B}}_0+\gamma [e_{r}+\alpha \Phi (p)]\}^2},\\ \dfrac{\partial H}{\partial e_m}= & {} \dfrac{\gamma e_{1/2}\alpha \Phi (p)p}{(e_{1/2}+G)^2}\cdot \dfrac{\partial G}{\partial e_{m}}, \quad \dfrac{\partial H}{\partial p}=1-\mu +\dfrac{\gamma \alpha \Phi (p)G}{e_{1/2}+G}+\dfrac{\gamma p}{e_{1/2}+G}\left\{ \alpha \Phi '(p)G+\dfrac{e_{1/2}\alpha \Phi (p)}{e_{1/2}+G}\cdot \dfrac{\partial G}{\partial p}\right\} , \end{aligned}$$

with $\Phi '(p)=-r_{0}\Phi (p)/[r_{0}p+\Gamma (p)]$ where $(\,)'$ means the derivative with respect to *p*. Then, the matrix of linearization around the equilibrium point ${\mathscr {E}}(P)$ is given by the Jacobian matrix in ${\mathscr {E}}(P)$,

$$\begin{aligned} J({\mathscr {E}}(P))=\begin{pmatrix} \dfrac{{\mathscr {B}}_0}{{\mathscr {B}}_0+\gamma \alpha \Phi (P)}&{}\quad 0\\ \dfrac{\gamma \alpha e_{1/2}{\mathscr {B}}_0{\mathscr {A}}P}{(e_{1/2}+E_{m})^{2}({\mathscr {B}}_0+\gamma \alpha {\mathscr {A}})}&{}\quad 1-\dfrac{\mu r_0P}{r_0P+K-{\mathscr {A}}P} \end{pmatrix}. \end{aligned}$$

Therefore, ${\mathscr {E}}(0)$ is uniformly asymptotically stable and ${\mathscr {E}}(\rho )$ is locally asymptotically stable.

In the second case, $0<\kappa \gamma <1$, we define the functions

$$\begin{aligned} F(e_{r},e_{m},p)= & {} (1-\kappa \gamma )[e_{r}+\alpha \Phi (p)],\\ G(e_{r},e_{m},p)= & {} \frac{{\mathscr {B}}_0[e_{m}+(1-\alpha )\xi _{c}\Phi (p)]}{{\mathscr {B}}_{0}+\gamma [e_{r}+\alpha \Phi (p)]},\\ H(e_{r},e_{m},p)= & {} \left\{ 1-\mu +\gamma [e_{r}+\alpha \Phi (p)]\frac{G(e_{r},e_{m},p)}{e_{1/2}+G(e_{r},e_{m},p)}\right\} p. \end{aligned}$$

Then, the discrete system is defined by equations $e_{r}^{k+1}=F(e_{r}^{k},e_{m}^{k},p^{k})$, $e_{m}^{k+1}=G(e_{r}^{k},e_{m}^{k},p^{k})$ and $p^{k+1}=H(e_{r}^{k},e_{m}^{k},p^{k})$ with $k\ge 0$ which has two equilibrium solutions: ${\mathscr {E}}(P)=(E_{r}(P),E_{m},P)$ where $E_{r}(P)=(1-\kappa \gamma )\alpha \Phi (P)/\kappa \gamma$, $E_{m}=\kappa {\mathscr {B}}_0 (1-\alpha )\xi _{c}/\alpha$ and $P\in \{0,\rho \}$. Then, $\partial F/\partial e_r=(1-\kappa \gamma )$, $\partial F/\partial e_m=0$, $\partial F/\partial p=(1-\kappa \gamma )\alpha \Phi '(p)$, $\partial H/\partial e_{r}=\gamma p G / (e_{1/2}+G)$,

$$\begin{aligned} \dfrac{\partial G}{\partial e_r}= & {} -\dfrac{\gamma {\mathscr {B}}_0[e_{m}+(1-\alpha )\xi _{c}\Phi (p)]}{\{{\mathscr {B}}_0+\gamma [e_{r}+\alpha \Phi (p)]\}^2}, \quad \dfrac{\partial G}{\partial e_m}=\dfrac{{\mathscr {B}}_0}{{\mathscr {B}}_0+\gamma [e_{r}+\alpha \Phi (p)]},\\ \dfrac{\partial G}{\partial p}= & {} \dfrac{{\mathscr {B}}_0[(1-\alpha )\xi _{c}({\mathscr {B}}_0+\gamma e_{r})-\alpha \gamma e_{m}]\Phi '(p)}{\{{\mathscr {B}}_0+\gamma [e_{r}+\alpha \Phi (p)]\}^2}, \quad \dfrac{\partial H}{\partial e_m}=\dfrac{\gamma e_{1/2}[e_{r}+\alpha \Phi (p)]p}{(e_{1/2}+G)^2}\cdot \dfrac{\partial G}{\partial e_{m}}, \end{aligned}$$

and

$$\begin{aligned} \dfrac{\partial H}{\partial p}=1-\mu +\dfrac{\gamma [e_{r}+\alpha \Phi (p)]G}{e_{1/2}+G}+\dfrac{\gamma p}{e_{1/2}+G}\left\{ \alpha \Phi '(p)G+\dfrac{e_{1/2}[e_{r}+\alpha \Phi (p)]}{e_{1/2}+G}\cdot \dfrac{\partial G}{\partial p}\right\} . \end{aligned}$$

The analysis of stability for the equilibrium solution ${\mathscr {E}}(0)$ is equivalent to that carried out in the first case. Thus, ${\mathscr {E}}(0)$ is uniformly asymptotically stable.

To analyze the stability of the equilibrium point ${\mathscr {E}}: = {\mathscr {E}}(\rho )$, we consider the characteristic polynomial of the linearization matrix around this point, $\tilde{F}(x)=x^3+ax^2+bx+c=0$, where

$$\begin{aligned} a= & {} -\left( \dfrac{\partial F}{\partial e_r}({\mathscr {E}})+\dfrac{\partial G}{\partial e_m}({\mathscr {E}})+\dfrac{\partial H}{\partial p}({\mathscr {E}})\right) ,\\ b= & {} \dfrac{\partial F}{\partial e_r}({\mathscr {E}})\left( \dfrac{\partial G}{\partial e_m}({\mathscr {E}})+\dfrac{\partial H}{\partial p}({\mathscr {E}})\right) +\dfrac{\partial G}{\partial e_m}({\mathscr {E}})\dfrac{\partial H}{\partial p}({\mathscr {E}})-\left( \dfrac{\partial F}{\partial p}({\mathscr {E}})\dfrac{\partial H}{\partial e_r}({\mathscr {E}})+\dfrac{\partial G}{\partial p}({\mathscr {E}})\dfrac{\partial H}{\partial e_m}({\mathscr {E}})\right) ,\\ c= & {} \dfrac{\partial F}{\partial p}({\mathscr {E}})\left( \dfrac{\partial G}{\partial e_m}({\mathscr {E}})\dfrac{\partial H}{\partial e_r}({\mathscr {E}})-\dfrac{\partial G}{\partial e_r}({\mathscr {E}})\dfrac{\partial H}{\partial e_m}({\mathscr {E}})\right) +\dfrac{\partial F}{\partial e_r}({\mathscr {E}})\left( \dfrac{\partial G}{\partial p}({\mathscr {E}})\dfrac{\partial H}{\partial e_m}({\mathscr {E}})-\dfrac{\partial G}{\partial e_m}({\mathscr {E}})\dfrac{\partial H}{\partial p}({\mathscr {E}})\right) . \end{aligned}$$

From the sign of the derivatives $a,\, c<0$ and $b>0$ are obtained. Thus, the necessary conditions of Jury’s criteria: (i) ${\tilde{F}}(1)>0$, (ii) ${\tilde{F}}(-1)<0$ and (iii) $|c|<1$, are met. Indeed, ${\tilde{F}}(1)=\kappa \gamma A_{1}A_{3}[1+(1-\kappa \gamma )A_{2}A_{3}]$, ${\tilde{F}}(-1)=-(1+|a|+b+|c|)$ and $|c|=(1-\kappa \gamma )(1-A_3)(1+\kappa \gamma A_1 A_2 A_3)$ where $A_{1}=\mu r_{0}\rho /(r_0\rho +K-{\mathscr {A}}\rho )$, $A_2=e_{1/2}/(e_{1/2}+E_{m})$ and $A_{3}=\alpha {\mathscr {A}}/(\kappa {\mathscr {B}}_0+\alpha {\mathscr {A}})$ with $0<A_{i}<1$ for $i\in \{1,2,3\}$. Note that $|c|<(1-A_3)[1-(\kappa \gamma )^2]<1$. In addition, the sufficient condition $1-c^2>|ac-b|$ is equivalent to $c^2-1<ac-b<1-c^2$. From (i), we have that $1-c^2-(ac-b)+(a-bc)>0$ and $1-c^2-(ac-b)-(a-bc)>0$. Adding these expressions, $ac-b<1-c^2$ is obtained. Whereas for analyzing the inequality $c^2-1<ac-b$ we consider the expression

19 $$\begin{aligned} c^2-ac+b&=  {} (1-\kappa \gamma )(1+\kappa \gamma A_{1}A_{2}A_{3})(1-A_3)\left\{ (1-\kappa \gamma )(1+\kappa \gamma A_{1}A_{2}A_{3})(1-A_3)\right. \nonumber \\&\left. -\, \left[ (1-\kappa \gamma )+(1-A_3)+1-A_{1}(\kappa \gamma +(1-\kappa \gamma )A_{2}A_{3})\right] \right\} +(1-\kappa \gamma )\nonumber \\&+\,(1-A_3)+1-A_1[\kappa \gamma (1-A_3)+(1-\kappa \gamma )^2 A_{2}A_{3}]-\left[ 1-(1-\kappa \gamma )(1-A_3)\right] . \end{aligned}$$

**Figure 3:**
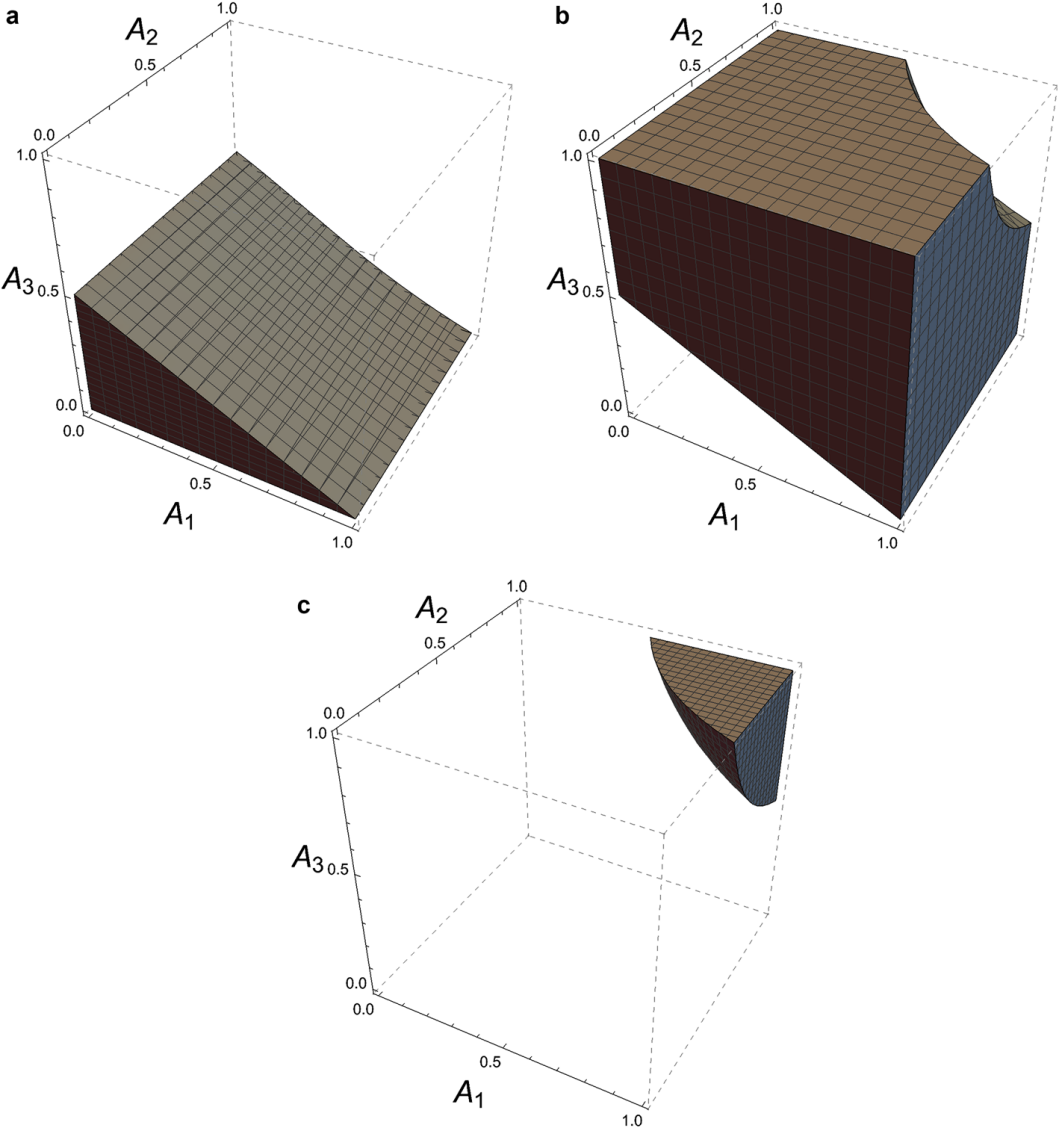
Disjoint regions of the unit cube $(0,1)^{3}$. (**a**) ${\mathscr {R}}_1$, (**b**) ${\mathscr {R}}_2$ and (**c**) ${\mathscr {R}}_3$.

Note that in Eq. (19), $\kappa \gamma$ is the amount that provides the greatest variability to this expression. Then, we define the function

$$\begin{aligned} g(x)&=  {} (1-x)(1+x A_{1}A_{2}A_{3})(1-A_3)\left\{ (1-x)(1+x A_{1}A_{2}A_{3})(1-A_3)-[(1-x)+(1-A_3)+1-A_{1}(x+(1-x)A_{2}A_{3})]\right\} \\&+(1-x)+(1-A_3)+1-A_1[x(1-A_3)+(1-x)^2 A_{2}A_{3}]-\left[ 1-(1-x)(1-A_3)\right] , \end{aligned}$$

for any $x\in [0,1]$. Now, we will show that $0<g(x)<1$ for any $x\in [0,1]$. The function *g* is a fourth degree polynomial $g(x)=B_{4}x^4+B_{3}x^3+B_{2}x^2+B_{1}x+B_0$ with coefficients

$$\begin{aligned} B_{4}&=  {} A_{1}^2 A_{2}^2 A_{3}^{2} (1-A_{3})^2>0,\\ B_{3}&=  {} A_{1}A_{2}A_{3}(1-A_3)[(1-A_1 A_2 A_3)(1-2A_3)-A_1],\\ B_{2}&=  {} -\{A_{1}[A_{2}A_{3}^2(1-A_{1}A_{2})(1-A_3)+1-A_{1}A_{2}A_{3}(1-A_{3})]+A_3(1-A_1)\}<0,\\ B_{1}&=  {} -A_{3}\{A_{1}A_{2}[1-A_{3}^2(1-A_{1}A_{2})]+A_{3}[2-(1+A_{1}A_{2})^2]\},\\ B_{0}&=  {} 1-A_{1}A_{2}A_{3}^2>0, \end{aligned}$$

where the values of $B_3$ and $B_1$ depend on the following three disjoint regions (see Fig. 3):

$$\begin{aligned} {\mathscr {R}}_1&=  {} \{(A_1,A_2,A_3)\in (0,1)^3: B_3\ge 0\wedge B_1<0\},\\ {\mathscr {R}}_2&=  {} \{(A_1,A_2,A_3)\in (0,1)^3: B_3<0\wedge B_1\le 0\},\\ {\mathscr {R}}_3&=  {} \{(A_1,A_2,A_3)\in (0,1)^3: B_3<0\wedge B_1>0\}. \end{aligned}$$

**Figure 4:**
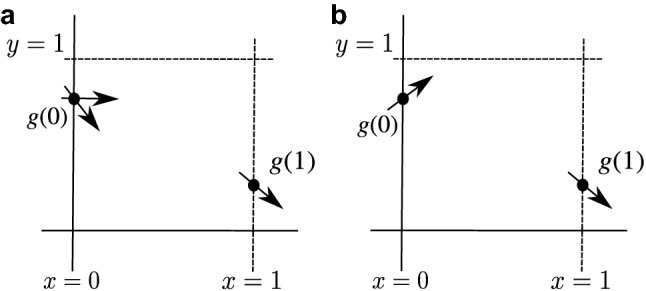
Graphical representation of some important properties of the polynomial *g* in the interval [0, 1]. (**a**) $$(A_1,A_2,A_3)\in {\mathscr {R}}_1\cup {\mathscr {R}}_2$$, and (**b**) $$(A_1,A_2,A_3)\in {\mathscr {R}}_3$$. The arrow on the axes $$ x = 0 $$ and $$x = 1$$ represent the sign of $$ g'(0)$$ and $$g'(1)$$ respectively.

Some important properties of the function *g* are: $g(0)=B_0\in (0,1)$, $g(1)=(1-A_1)(1-A_3)>0$, $g(0)-g(1)=A_1(1-A_3)+A_3(1-A_1A_2A_3)>0$ which implies that $0<g(1)<g(0)<1$. Also, $g'(0)=B_1$ and $g'(1)=-\{(1-A_3)(2A_1+A_3)+A_3(1-A_1 A_2)^2+A_1 A_2 A_3^{2}(A_1+3-A_3)\}<0$ (see Fig. 4).

Therefore, by the method of Descartes, we have the five admissible cases which are presented in Table 1. On the one hand, in the cases $C_1$, $C_3$, $C_4$ and $C_5$, $g'$ has there is only one positive real root ${\overline{x}}={\overline{x}}(A_1,A_2,A_3)$ where $(\, )'$ means the derivative with respect to *x*. Note that ${\overline{x}}>1$, because otherwise there would be at least one additional zero of $g'$ on the interval (0, 1) for the basic properties are met. Then, the function *g* is a positive and decreasing function for any $x\in [0,1]$ with maximum value $g(0)=B_0<1$. On the other hand, in case $C_2$, $g'$ has one positive real root ${\overline{x}}\in (0,1)$ at which the polynomial *g* has a local maximum. Taking $g({\overline{x}})$ to define a function $f(A_1,A_2,A_3)$ for $(A_1, A_2, A_3)\in {\mathscr {R}}_3$, we have that $0<f(A_1,A_2,A_3)<1$ if and only if $(A_1,A_2,A_3)\in {\mathscr {R}}_3$ (using the application Mathematica). Thus, in all cases, $0<g(x)<1$ for any $x\in [0,1]$, which implies $c^2-ac+b<1$ and hence $c^2-1<ac-b$. Therefore, ${\mathscr {E}}(\rho )$ is locally asymptotically stable. $\square$

**Table 1:** Admissible cases given by the method of Descartes applied to the polynomial *g* assuming that its coefficients $B_{i}$ with $i\in \{0,1,2,3,4\}$ take the values according to $(A_1,A_2,A_3)\in (0,1)^{3}$. Each row corresponds to the case in which the signs of the coefficients of *g* are as indicated depending on the region ${\mathscr {R}}_{j}$ with $j\in \{1,2,3\}$.

Case	$B_4$	$B_3$	$B_2$	$B_1$	$B_0$	Region
$C_1$	+	+	−	−	+	${\mathscr {R}}_1$
$C_2$	+	−	−	+	+	${\mathscr {R}}_3$
$C_3$	+	−	−	−	+	${\mathscr {R}}_2$
$C_4$	+	0	−	−	+	${\mathscr {R}}_1$
$C_5$	+	−	−	0	+	${\mathscr {R}}_2$

### Optimal strategy

**Figure 5:**
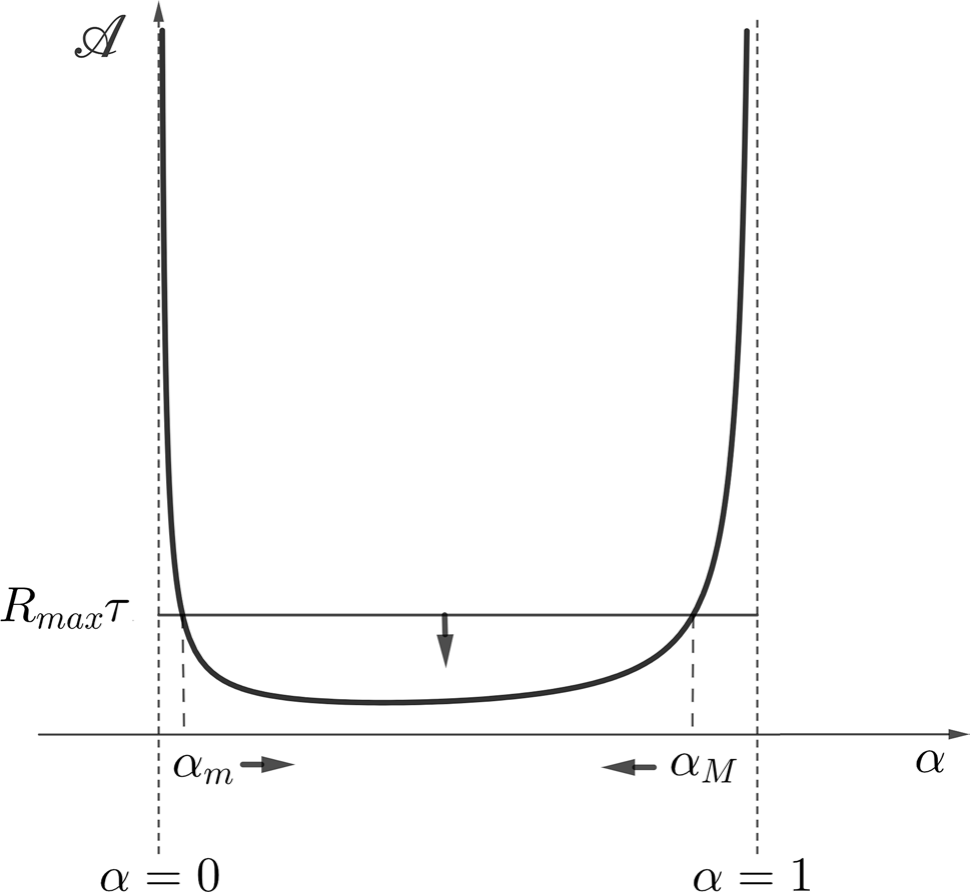
The individual consumption in the long term versus allocation strategy towards reproduction. Note that the size of the persistence range reduces with decreasing length of the cycle $\tau$ or of the maximum rate of consumption $R_{max}$, which is indicated by the respective arrows. In addition, ${\mathscr {A}}={\mathscr {A}}(\alpha )$ is a convex function, which implies that there is a unique allocation strategy, into the persistence range, that minimizes individual consumption in the long term.

The validity of the condition ${\mathscr {U}}> 1$ depending on $\alpha$ establishes a range of allocation strategies $(\alpha _m, \alpha _M)$ in the which the persistence of the population is guaranteed (see Fig. 5). Thus, $\alpha \in (\alpha _{m}, \alpha _{M})$ if and only if ${\mathscr {U}}> 1$, where $\alpha _{m}$ and $\alpha _{M}$ are the limits of this range such that ${\mathscr {U}} = 1$ is obtained.

**Figure 6:**
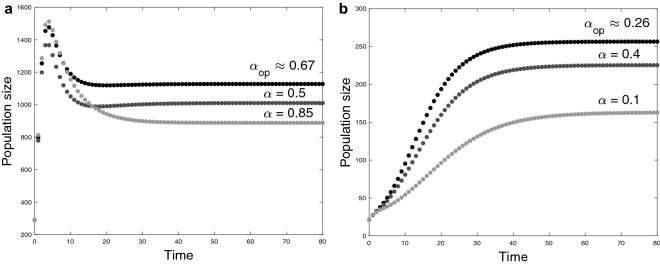
Comparison of the long-term behavior of the population size after the reproductive season (population component of the system (6)) using various allocation strategies towards reproduction. (**a**) We consider the parameter set $\eta =(2,1,1,0,200,2,2,1/3,0.1,0.39,\alpha ,0.2)$ with $\alpha \in \{0.5,0.67,0.85\}$ and (**b**) $\eta =(2,1,0.5,0,400,1,2,1/3,0.1,0.63,\alpha ,0.2)$ with $\alpha \in \{0.1,0.26,0.4\}$. In both cases, the constant of fertility costs is described by $\kappa =(1+\gamma )^{-1}$. In addition, note that in (**a**) $\Delta =2/3>\delta \approx 0.165$ and (**b**) $\Delta =1/3<\delta \approx 2.718$.

In this persistence range we find a unique allocation strategy described by

$$\begin{aligned} \alpha _ {op} =\left( 1+\sqrt{\dfrac{e_{1/2}\xi _{c}^{-1}}{\kappa {\mathscr {B}}_0}}\,\right) ^{-1}, \end{aligned}$$

which maximizes the population abundance and minimizes the individual consumption in the long term (see Fig. 6). This allocation strategy satisfies $\rho '(\alpha _ {op}) = 0$ if, and only if, also satisfies ${\mathscr {A}}'(\alpha _ {op}) = 0$ where $(\,)'$ means the derivative with respect to $\alpha$ and

$$\begin{aligned} {\mathscr {A}}'(\alpha )=\dfrac{\mu }{{\mathscr {B}}_0}\left\{ \dfrac{e_{1/2}\xi _{c}^{-1}}{(1-\alpha )^2}-\dfrac{\kappa {\mathscr {B}}_0}{\alpha ^2}\right\} . \end{aligned}$$

In addition, the strategy $\alpha _ {op}$ is dependent on the parameters associated with both the costs of fertility ($\kappa$ and ${\mathscr {B}}_0$) and survival ($\xi _{c}$ and $e_{1/2}$), so that if the fertility costs $\Delta :=\kappa {\mathscr {B}}_0$ are greater than the maintenance ratio $\delta :=e_{1/2}/\xi _{c}$, the allocation is to favor reproduction, i.e., $\alpha _{op}>0.5$ (see Fig. 6a). Otherwise, the allocation is to favor maintenance, i.e., $1-\alpha _{op}\ge 0.5$ (see Fig. 6b).

## Discussion

By studying the dependence of the population abundance in seasonal environments on strategies of energy allocation to life-history functions, we have proposed a consumer–resource model built from the division of the annual cycle (into a reproductive season and a non-reproductive season) and the investment of the resource, as an incoming energy flow to reproduction and maintenance.

In stable environments that have a constant amount of resources (or that exhibit only minimal variations) between consecutive annual cycles, long-term persistence is a consequence of negative feedback between the increase in population size and available resources^52^. Thus, our model showed that long-term trends of population size (i.e., extinction and persistence) depends on the per capita consumption of the resource. Such behaviors are differentiated through a threshold value that admits of a representation as the per capita consumption of the resources in the long term.

The differentiation of dynamic behaviors that emerge from biological models is often done through an amount that acts as a threshold value (e.g.,^53,54^). Specifically, in ecological populations with age structure, population dynamics are determined by the net reproductive rate ${\mathscr {R}}_0$^5,49^ that determines the population growth and the potential self-replacement when ${\mathscr {R}} _0 \ge 1$. On the contrary, when ${\mathscr {R}} _0 <1$, the long-term dynamics results in extinction.

Our approach assumes that the assigned resource is an energy source for individuals, invested in life-history functions (e.g., reproduction and maintenance) that determine the population growth^52,55^. This corresponds with the outcome of interconnections between food, allocation and life-history^56^ that through our model allows us to find classical behaviors for the size of the population, which correspond to extinction and persistence, and in response to a density-dependent mechanism. A wide literature has shown there is an increase (generally, with a maximum value) of the per capita population growth rate (*r*) when there is an increase in food availability at both the population and individual levels^52,55,57–62^. Additionally, it shows that *r* is negatively related to the population density (*D*), (e.g., $r\propto D^{-1}$) which allows concluding that there is a negative relation between the per capita availability of resources (*F*) and *D* (e.g., $F\propto D^{-1}$).

Our results establish that the individual scale is important for population persistence. Individual consumption $C_{I}^{\infty }(\rho )$ is the quantity that collects the main ecological parameters of the life-history assumed in this model. The parameter that causes the greatest variability in $C_{I}^{\infty }(\rho )={\mathscr {A}}$ determines the allocation of resources towards reproduction. For this parameter, we have found the range of values that allow long-term population persistence. There is a unique strategy $\alpha _{op}$ which implies a maximum population size $\rho (\alpha _{op})$ and a minimum consumption per individual ${\mathscr {A}}(\alpha _{op})$ in the long term, inversely related quantities since $\rho (\alpha _{op})\cdot {\mathscr {A}}(\alpha _{op})\propto K$. This optimal strategy depends on the parameters associated with fertility and survival costs, $\alpha _{op} = \alpha _{op}(\kappa , {\mathscr {B}}_0,e_{1/2},\xi _{c})$. Although our modeling approach considers allocation strategies represented by a parameter and not by a time-dependent variable state or an environmental measure, the optimal allocation strategy $\alpha _{op}$ shows variability, being plastic, either when the fertility cost and/or the maintenance ratio varies. Indeed, when the maintenance ratio decreases ($\delta = e_{1/2}/\xi _{c} \rightarrow 0$) the individuals increase their survival due to (i) a decrease in the maintenance cost factor $\xi$ ending the non-reproductive season with higher maintenance energy $E_{m}(n \tau )$ or (ii) a decrease in the amount of $e_{1/2}$ which implies that only a little maintenance energy $E_{m}(n\tau ^+)$ is needed to guarantee the individual’s survival. In this case, the optimal allocation strategy favors the reproductive process, as Fischer et al. (2009,2010)^50,51^ point out. Conversely, the optimal allocation strategy favors the maintenance process.

In this study, constant allocation strategies establish the persistence and stability of the population when the threshold value is ${\mathscr {U}}>1$. This result differs from those found by Akhmetzhanov et al. (2011), who established that the strategies of constant allocation lead to population extinction due to a gradual loss or over-exploitation of the resource. This difference may be related to the different mathematical representations used to describe the dynamics in the reproductive and non-reproductive seasons, where we highlight the functional response chosen to describe the predator–prey relationship (ratio-dependent vs. linear prey-dependent), and the variability in the resource density available for consumption at the beginning of each non-reproductive season (constant resource vs. cumulative resource). We propose that these and other differences should be studied in a general modeling framework that incorporates resource abundance, internal energy, and consumer abundance as state variables, namely, a consumer–resource and energetic mathematical model that incorporates the individual-level in consumer–resource interactions.

We showed that the consumer population’s ecological dynamics is limited both by the quality or quantity of food and by the design of the acquisition and energy allocation process. Consequently, the usefulness of our model lies in studying the occurrence of the potential behavior outcomes in the long term for a consumer population as a function of parameters of ecological significance. In the proposed mathematical formulation, the reproductive season’s ecological dynamics are relevant, allowing extrapolating some of the conclusions obtained. The main reproductive trade-off related to energy allocation is the offspring size and offspring mass. The results of Veloso and Bozinovic (2000)^63^ show that with the same reproduction effort, it is possible to obtain numerous small-size offspring or a few large-size offspring, due to the positive relations between reproductive effort, the organism’s body mass, and the resting metabolic rate. From our model, an increased reproductive effort leads to a lower reproduction energy available after the reproductive season, which implies a lower long-term population size. Due to the negative relation between individual consumption and population size, it is possible to conjecture that the optimal life-history strategy results in a small number of large-size offspring. The organisms have a finite amount of resources that are invested in multiple tasks. When the availability of resources is compromised, organisms often respond by making physiological adjustments (e.g., the resting metabolic rate), allowing the allocation of resources to other vital processes^63,64^. Peña et al. (2020) has shown that stochastic food deprivation produces a compromise between energy expenditure rates and locomotion activity performed by individuals, describing it as a paradoxical result. However, we can hypothesize that a lower individual consumption together with a higher maintenance cost ($\xi \rightarrow 1$) implies that this compensation is mediated by an optimal energy allocation, favoring naturally the maintenance activities, sustaining the locomotor (or exploratory) behavior.

The model proposed in the present paper has made several assumptions that make it both generally and mathematically accessible. First, all individuals in the population have common traits, such as the allocation strategy towards reproduction. Our approach is based on the theory of life-history that is shared and incorporated by numerous investigations^24^. Therefore, an interesting extension of our model arises precisely by incorporating the variability of traits through phenotypic distributions. Second, energetic resources are assigned to reproductive and maintenance processes, with the latter concentrating on the survival and/or growth of individuals during the annual cycle. Specifically, body size as a measure of an individual’s growth has been related to life-history parameters^28,65,66^ and birth rates and mortality^3^. We conjecture that incorporating body size would allow us to analyze the consumer–resource dynamics in animals that exhibit determined or indeterminate growth^9,31^. Third, for mathematical simplicity, a constant population size was assumed over the non-reproductive season for obtaining an autonomous system, i.e., independence of the temporal variable. The reduction in population size during the non-reproductive season, by assuming $\lambda > 0$, implies an increase in per capita resource consumption, which reduces the set of strategies that establish the persistence of the population. Therefore, in the general case, the size of the persistence range $L=\alpha _{M}-\alpha _{m}$ depends on the population size. However, the long-term dynamic behaviors remain unchanged. Fourth, at the end of each reproductive season, the individuals that enter the population do not differ by age from the surviving individuals (except in the case $\mu = 1$). Such differentiation would permit an advance towards a reformulation of our model with an age structure, incorporating physiological differences between individuals of different groups. These points pose new challenges for future research.
